# SWI and CTP fusion model based on sparse representation method to predict cerebral infarction trend

**DOI:** 10.3389/fnins.2024.1360459

**Published:** 2024-06-20

**Authors:** Guoqing Wu, Hao Wang, Xiaojun Ma, Huanyin Li, Bin Song, Jing Zhao, Xin Wang, Jixian Lin

**Affiliations:** ^1^Department of Electronic Engineering, Fudan University, Shanghai, China; ^2^Department of Radiology, Minhang Hospital, Fudan University, Shanghai, China; ^3^Department of Laboratory Medicine, Minhang Hospital, Fudan University, Shanghai, China; ^4^Department of Neurology, Minhang Hospital, Fudan University, Shanghai, China; ^5^Department of Neurology, Zhongshan Hospital, Fudan University, Shanghai, China

**Keywords:** SWI, CTP, radiomics, sparse representation, infarction trend

## Abstract

**Objective:**

SWI image signal is related to venous reflux disorder and perfusion defect. Computed tomography perfusion (CTP) contains perfusion information in space and time. There is a complementary basis between them to affect the prognosis of cerebral infarction.

**Methods:**

Sixty-six patients included in the retrospective study were designated as the training set. Effective perfusion indicator features and imaging radiomic features of the peri-infarction area on Susceptibility weighted imaging (SWI) and CTP modality images were extracted from each case. Thirty-three patients from the prospectively included group were designated as the test set of the machine learning model based on a sparse representation method. The predicted results were compared with the DWI results of the patients’ 7–10 days review to assess the validity and accuracy of the prediction.

**Results:**

The AUC of the SWI + CTP integrated model was 0.952, the ACC was 0.909, the SEN was 0.889, and the SPE was 0.933. The prediction performance is the highest. Compared with the value of AUC: the SWI model is 0.874, inferior to the performance of the SWI + CTP model, and the CTP model is 0.715.

**Conclusion:**

The prediction efficiency of the changing trend of infarction volume is further improved by the correlation between the combination of the two image features.

## Introduction

Ischemic stroke is a severe neurological disease and a leading cause of disability and death worldwide (Li and Zhang, 2019; Wang et al., 2020). The most important treatment for acute ischemic stroke (AIS) is recanalization of the occluded vessel within a strict time window to salvage the ischemic penumbra (PEN) (Miteff et al., 2009; Shuaib et al., 2011). Over the past 40 years, imaging studies on ischemic penumbra identification have greatly extended the time window for reperfusion therapy and better predicted post-reperfusion clinical outcomes relative to treatment time by delineating the infarct core volume, expanding the cohort of patients benefiting from reperfusion therapy who were excluded due to time constraints (Bivard et al., 2015; Kawano et al., 2017). This provided a better regimen for selecting AIS patients for reperfusion therapy, perfectly interpreting “tissue is as important as time” beyond time is brain (Ermine et al., 2021).

Recent studies on the heterogeneity of the ischemic penumbra evaluated high-risk ischemic penumbra in patients with acute ischemic stroke (AIS) by using infarct core growth rate (ICGR). Infarct core growth rates were calculated by dividing the core volume by the time between onset and image acquisition, with units of mL/h (Lin et al., 2021; Sarraj et al., 2021). ICGR was highly correlated with final infarct volume and degree of neurological impairment, indirectly revealing the evolution process of ischemic penumbra to infarction, which largely depends on collateral circulation status (Renú Jornet et al., 2020). AIS patients with faster infarct core growth generally have poorer collateral circulation and benefit more from reperfusion therapy (Lin et al., 2021). Slow progressors represent small infarct cores and better collateral circulation. However, collateral circulation is unstable and may either maintain a relatively good state or deteriorate, becoming part of the infarct core (Vagal et al., 2018). Therefore, rapidly progressive patients can be screened by ICGV for timely reperfusion therapy to benefit, but clear clinical decisions cannot be made regarding whether slowly progressive patients need reperfusion therapy (Rocha et al., 2019). If subsequent infarction changes could be predicted, those expected to have infarct enlargement could receive timely intervention, while those not requiring reperfusion could avoid the risks of thrombectomy.

Changes in the core of cerebral infarction are a dynamic process due to the local hemodynamic impairment that occurs around the infarction and changes spatially over time. Therefore, the pathophysiological process of brain infarction contains both temporal and spatial dimensions. By extracting imaging features from early perfusion images around the VOI region, features about lesion growth dynamics can be captured, and tissue outcomes predicted.

Simultaneous perfusion techniques can provide functional and circulatory information about the pia mater and secondary collateral pathways by adding a temporal dimension (Keedy et al., 2012). However, most studies have focused on time-based perfusion maps, suggesting that patients with good collateral circulation have less flow delay (Campbell et al., 2013) and greater perfusion volume (Marks et al., 2014; Vagal et al., 2016). The downside of these perfusion-based global modeling approaches is that differences in affected tissue location have no relevant effect on tissue outcomes and are performed independently for each VOI, still ignoring the valuable spatial information that perfusion can provide.

SWI is a high-resolution T2* MRI technique with a three-dimensional gradient-echo sequence sensitive to magnetization rate differences (Liu et al., 2017). Metabolic changes in hypo-perfused brain tissues (Haacke et al., 2009; Tsui et al., 2009) can be studied by acting on the sensitivity of paramagnetic materials.

Studies have shown that SWI also plays an essential role in predicting the prognosis of patients with acute cerebral infarction (Chen et al., 2015; Mundiyanapurath et al., 2015). Decreased flow and increased OEF in ischemic areas of the brain lead to more deoxyhemoglobin, resulting in prominent venous hypo-signal on SWI images, the so-called Venous Protrusion Syndrome (PVS) described in the MRI-SWI sequence (Park et al., 2014; Polan et al., 2015). Studies have shown that asymmetric cortical venous low signal correlates with increased MTT and TTP in perfusion parameters and is positively correlated with the degree of perfusion defects in the ischemic core, which can be used as a marker of the ischemic penumbra (Kao et al., 2012). The more prominent veins, the larger the volume of hypoperfusion tissue, and it is related to early changes in NIHSS score after acute treatment (Jensen-Kondering and Bohm, 2013).

In conclusion, there are complementarities between the two in detecting blood perfusion. To further improve the model’s prediction performance, the two are fused to build a comprehensive model. We use the sparse representation method to screen and clarify the image markers affecting the prognosis of cerebral infarction and establish the prediction model of cerebral infarction prognosis.

## Methods

### Research object

The study was registered in Chinese Clinical Trial Registry (Registration number: ChiCTR2100045753). Ethical approval for this study was obtained from the institutional review board at Minhang Hospital, Fudan University, Shanghai, China. The study was performed by the ethical standards as laid down in the 1964 Declaration of Helsinki and its later amendments or comparable ethical standards. The need for consent for this study was waived by the institutional review board (the institutional review board at Minhang Hospital, Fudan University, Shanghai, China) due to its design.

### Case collection

#### Enrollment criteria

All enrolled patients sought medical help within 3 days of the onset of symptoms. They exhibited newly surfaced neurological deficit symptoms and signs that align with the clinical presentation of acute ischemic stroke. After admission, all patients underwent a non-contrast CT scan immediately to rule out intracranial hemorrhage and other etiologies. All met the diagnostic criteria for acute cerebral infarction. All were diagnosed by imaging and neurological physical examination, such as cranial CT or cranial MRI (the cranial CTP was done immediately after the first cranial MRI), with NIHSS scores of 0–25 and within 72 h of onset.

#### Treatment approach

For patients suitable for endovascular thrombectomy or intravenous thrombolysis, we followed the guidelines to initiate treatment as soon as possible without delay for imaging studies. Upon admission, patients were provided with timely standardized treatment following the guidelines—including but not limited to antiplatelet or anticoagulant therapy—depending on their individual medical condition.

#### Retrospective analysis

Sixty-six inpatients with acute cerebral infarction who visited the Department of Neurology, Minhang Hospital, Fudan University, from January 2018 to June 2021.

**Figure 1:**
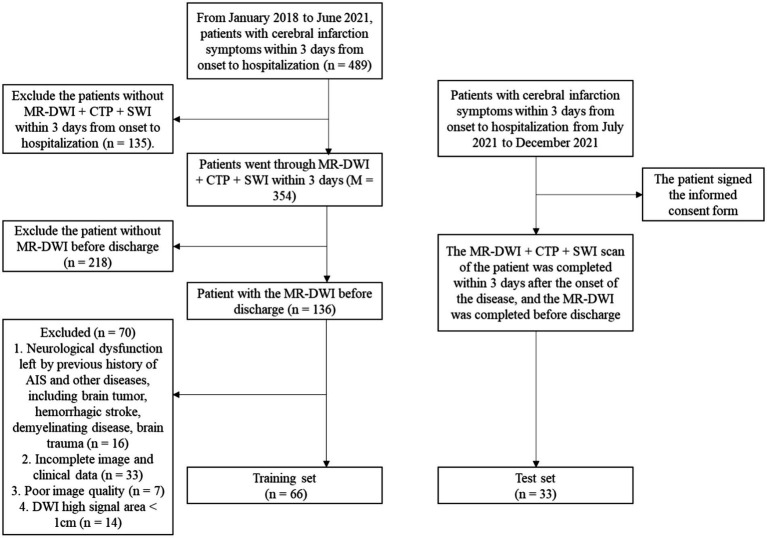
Flow chart of enrolled patients in the brain parenchyma-related model study.

During the study period, 489 patients with cerebral infarction admitted within 3 days of onset were collected, 135 patients who did not have MR-DWI + CTP + SWI within 3 days were excluded, and 218 patients who did not have a repeat MRI-DWI were excluded. 136 patients completed two MRIs and had a combination of previous cerebral infarction or other diseases causing neurological deficits, including brain tumors, hemorrhagic stroke, demyelinating disease, and traumatic brain injury (*n* = 16); incomplete imaging and clinical data (*n* = 33); poor image quality (*n* = 7); and a maximum diameter of DWI high signal area <1 cm (*n* = 14). Ultimately, 66 patients were included in this study (Figure 1).

#### Prospective validation

After the model was established, 33 inpatients with acute cerebral infarction admitted to the Department of Neurology in our hospital from July 2021 to December 2021 were prospectively included and signed the consent form (Figure 1).

Sixty-six patients from this retrospective study were assigned to the training set, and 33 patients from prospective enrollment were assigned to the test set. The flow chart of all patients enrolled in the study is shown in Figure 1. It can be seen from the data collection process that although 489 patients were included in the initial stage of this study, 99 patients were eventually included in the study due to various reasons such as incomplete MRI examination modes, missing follow-up data, low imaging quality, and patient factors.

### Imaging examination and biochemical examination

#### Imaging examination

To ensure the examination did not interfere with those eligible for reperfusion therapy within the time window. The examinations were typically scheduled 24 h after admission and within 3 days.

#### Cranial MRI parameters

All MRI examinations were performed using a single 3.0 T magnetic resonance scanner (umr780, Shanghai Lianying) to maintain consistency across the study, using a commercial 24-channel head and neck coil. The MRI scheme is as follows:

T1 – weighted fast spin-echo (FSE) sequence [repetition time (TR)/echo time (TE) = 2048/11.96 ms; Turning angle (FA) = 135°; Slice thickness/gap = 5/1.5 mm; Bandwidth = 180 Hz/PX; FOV = 230 × 200 mm^2^; Acceleration factor = 2].

T2 weighted FSE sequence (TR/TE = 4107/88.2 ms; FA = 145°; slice thickness/gap = 5/1.5 mm; bandwidth = 180z/PX; FOV = 230 × 200 mm^2^; Acceleration factor = 2).

T2 – FLAIR sequence (TR/TE = 7500/96.66 ms; FA = 150°; slice thickness/gap = 5/1.5 mm; bandwidth = 220 Hz/PX; FOV = 230 × 190 mm^2^).

SWI sequence (TR/TE = 30.2/20 ms; FA = 15°; slice thickness/gap = 2/1 mm; bandwidth = 130 Hz/PX; FOV = 224 × 190 mm^2^).

DWI adopts single-shot echo planar imaging (EPI) sequence (TR/TE = 2800/75.4 ms; FA = 90°; slice thickness/gap = 5/1.5 mm; bandwidth = 1790 Hz/PX; FOV = 230 × 220 mm^2^). 2 *b* values (0 and 1000s/mm^2^).

#### Parameters of cranial CTP examination

For the CTP examination, the same Siemens Somatom Force CT scanner, which is the third generation of dual-source force CT, was used for all scans. After the patient’s head was fixed in the supine position, the cranial CT scan was first performed with a tube voltage of 120 kv, automatic milliamperes, and a layer thickness of 5 mm. Fifty milliliters of Uvexan (370 mg/mL) was injected by a high-pressure syringe through the elbow vein using a 20G puncture needle. Physiological saline 20 mL, injection rate 6 mL/s, 5 s delay after injection to start CTP scan. The scanning range was from the level of the carotid bifurcation to 224 mm at the cranial apex. 1.5 s for a single scan, 26 consecutive dynamic scans, and 26 volumetric data were obtained, and the total scanning time was 39.39 s. The layer thickness of each sequence was 1 mm for thin-layer reconstruction.

#### Biochemical examination

The patient shall complete relevant laboratory examinations within 24 h after admission, including three routine examinations of hematuria and stool, blood glucose, glycosylated hemoglobin, D-dimer, four coagulation tests, renal function, low-density lipoprotein, homocysteine, fasting blood glucose, etc.

### Clinical evaluation

#### Severity assessment of neurological symptoms

NIHSS scores were recorded on admission and before discharge (about 7–10 days), as well as the mRS score scale for follow-up evaluation at about 90 days after onset.

### Feature extraction method based on image patch sparse representation

We first expanded the DWI segmented image in the previous section by 20 pixels outside the lesion area and matched the segmentation results of SWI images on the corresponding interface. Then, 513 gray features, texture, and wavelet features are extracted from the SWI image, as described in the Supplementary methods.

### Collection and formation of perfusion indicators

#### Time-density curve

**Figure 2:**
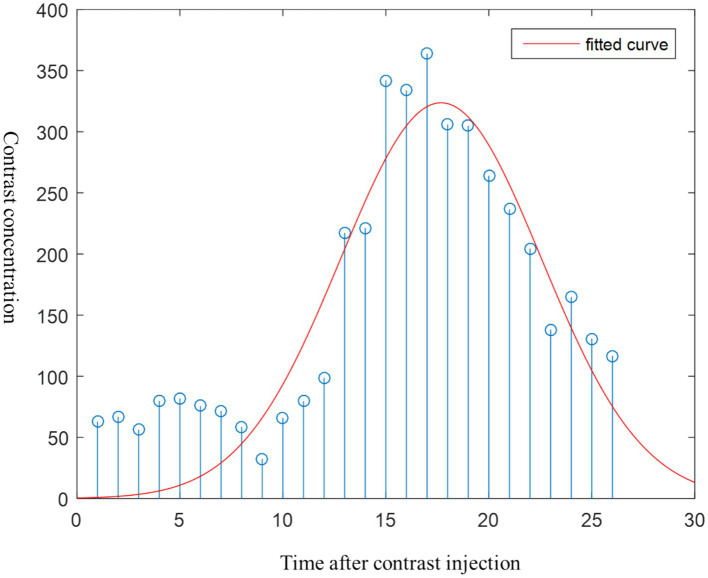
Fitting time density curve by voxel grayscale.

The time-density Curve (TDC) of patients were obtained from the CTP image, as described in the Supplementary methods. Figure 2 shows Gaussian fitting on the TDC curve of one patient’s data at the one-pixel point.

### Reconstruction of a pseudo color image of perfusion index

**Figure 3:**
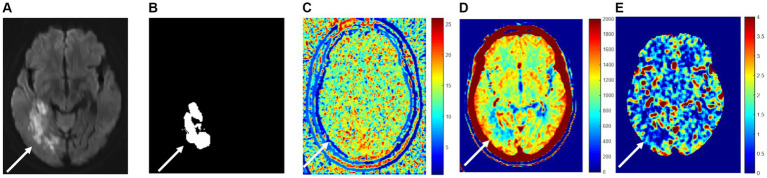
Perfusion indexes TTP, CBF, and CBV are blood flow parameters for pixel numerical imaging. **(A)** True cerebral infarction location; **(B)** the segmented area; **(C)** TTP; **(D)** CBV; **(E)** CBF.

According to the time density curve of each pixel and the definitions of TTP, CBF, and CBV, TTP, CBF, and CBV pseudo-color images are constructed (Figure 3).

Figure 3A illustrates the area of high signal intensity on DWI, representing the true infarction core. Figure 3B depicts the area segmented by extending approximately 2–3 mm beyond the high signal intensity region on DWI. Figures 3C–E shows peak time TTP pseudo-color map, relative cerebral blood volume CBV pseudo-color map, and relative cerebral blood flow CBF pseudo-color map at the same level, respectively. It can be visualized from the figure that the infarction and the surrounding area blood flow changed because of the infarction area blood vessel blockage, so the peak time is relatively long, and the infarction area value in Figure 3C is relatively large because the infarction area blood vessel blockage, so the infarction area cerebral blood volume and flow are relatively small, the infarction area value in Figures 3D,E is relatively small.

### Feature extraction of perfusion index

High-throughput features of infarction and surrounding areas (the segmented area in Figure 3B) were extracted from CBV images, CBF images, and TTP images, as described in the Supplementary methods.

### Sparse representation feature selection

After high-throughput feature extraction, there may be a vast amount of feature redundancy. On the one hand, these redundant features added a lot of computation to the subsequent classification and recognition. On the other hand, they could also harm recognition accuracy. Therefore, it is essential to screen out the features with high resolution. The feature selection is conducted by establishing a sparse representation feature selection model. The formula for the sparse representation coefficient is Equation 1 as follows:


(1) $$\hat{w}=\underset{w}{argmin}l-Fw_{2}^{2}+\eta w_{0}$$

Where m is the number of training samples, $l\in R^{m}$ is the label of training samples, $F={\left[f_{1},f_{2}\cdots f_{m}\right]}^{T}\in R^{m\times 2K}$ is the feature set of training samples. η is the sparse representation control parameters. The absolute values of the elements in sparse representation coefficient w correspond to the importance of features. When w is obtained, a small number of features with high importance to classification can be selected to sort its absolute value.

### Imaging radiomics classification prediction model

Finally, the radiomic features of the peri-infarct region of SWI + CTP were extracted from each case. The volume of the infarct core area was obtained, and the images before and after treatment were compared. The difference was used as the grouping basis. The patients were divided into the reversal group and the enlargement group.

The increased or decreased infarction core volume was converted into a machine learning binary classification problem, and a sparse representation classifier based on non-parametric training was established for classification prediction. The sparse representation classifier first uses sparse coding and reconstruction to calculate the distance between the sample to be tested and each class of samples in the training set. It then judges the class classification process of the test sample according to the K-proximity criterion. Specifically, it can be expressed as Equations 2:


(2) $$\hat{\beta }=\underset{\beta }{argmin}||\bar{f}-\bar{F}\beta |{|}_{2}^{2}+\gamma ||\beta |{|}_{0}$$

where $\bar{f}$ denotes the features of the samples to be tested after feature selection, $\bar{F}=\left[{\bar{F}}_{1},\cdots {\bar{F}}_{c}\cdots {\bar{F}}_{C}\right]$ is the training sample feature set, and C is the total number of sample classes. For the binary classification problem here, C=2 . γ is the sparse representation control parameter. When the sparse representation coefficient $\hat{\beta }$ is obtained, the distance between the samples to be tested and each class of samples in the training set is calculated, i.e., the representation residuals of each class is Equations 3 as follows:


(3) $$r_{c}\left(f\right)=\bar{f}-\bar{F}\delta_{c}\left(\hat{\beta }\right),c=\left[1,2,\cdots ,C\right]$$

where $\delta_{c}\left(.\right)$ denotes the coefficient corresponding to the selection of the $c_{th}$ classes of features. The class with the smallest residual is the class of the test sample, so the final class of the sample to be tested is $Id\left(\bar{f}\right)=\underset{c}{argmin}r_{c}\left(\bar{f}\right)$ .

### Imaging radiomics model validation

After the model was established, 33 inpatients with acute cerebral infarction who visited our neurology department from July 2021 to December 2021 were prospectively included. The imaging radiomics features of the patients were extracted and screened by the model for inclusion in the prediction model. The predicted results were compared with the DWI results of the patients’ 7–10 day review to assess the validity and accuracy of the prediction.

### Statistical analysis and model evaluation

#### Baseline variable statistics

All grouped data were tested for Shapiro–Wilk normality by the shaporo.test () function in the R language. Variables such as demographics, clinically relevant indicators, and experimental examinations were used using the *tableone* package in R^1^ to compare differences between groups with different infarct change trend, as well as training set and test set differences. Age, sex, NHISS score, mRS score, infarct volume, and DWI-ASPECTS score were non-normally distributed continuous variables expressed as median (quartiles) and were compared between the two groups using the Wilcoxon test. MR examination time from the onset and laboratory tests were normally distributed as continuous variables and were compared between the two groups using the *t*-test, all expressed as mean (standard deviation). Risk factors and thrombolysis are classified variables, expressed as counts (percentage), and the fisher’s exact test was used when comparing the two groups. *p* < 0.05 was considered statistically significant.

#### Predictive model statistics

MATLAB software was used to establish four prediction models by sparse representation: DWI imaging-based radiomics model, CTP imaging-based radiomics model, SWI imaging-based radiomics model, and DWI + CTP + SWI imaging-based radiomics model.

Use the *evalmod* function in the *precrece* package of R language to calculate the basic evaluation index for the specified model. It includes accuracy (ACC), specificity (SPE), sensitivity (SEN), error (ERR), precision (precision), Matthews correlation coefficient (MCC), and *F*-score. The scatter diagram between normalized rank values was used as the reference for selecting the truncation value, as described in the Supplementary methods.

## Results

### Clinical features

The patients were grouped according to DWI reversal and DWI high signal enlargement (DWI enlargement for short), and the statistics of clinical index-related variables were conducted. Univariate analysis showed that the following variables were significantly correlated with the changing trend of infarct volume: the previous history of cerebral infarction (*p =* 0.023), low-density lipoprotein (*p =* 0.028), DWI-ASPECT score (*p =* 0.026), NIHSS score at discharge (*p =* 0.007), NIHSS score change during hospitalization (*p* < 0.001), mRS score at discharge (*p =* 0.001), and mRS score at 90 days (*p =* 0.001). There was no difference (ΔmRS) between the two (*p =* 0.423). In addition, hypertension (*p =* 0.138), diabetes (*p =* 1), hyperlipidemia (*p =* 1), coronary heart disease (*p =* 0.235), smoking (*p =* 0.672), drinking (*p =* 1), homocysteine (*p =* 0.374), glycosylated hemoglobin (*p =* 0.647), fasting blood glucose (*p =* 0.816), serum creatinine (*p =* 0.97), urea (uric acid), uric acid (AC), international normalized ratio (*p =* 0.165), fibrinogen (*p =* 0.51), platelet (*p =* 0.582), Baseline NIHSS score at admission (*p =* 0.816) (Tables 1, 2). Nineteen patients in this cohort received intravenous thrombolysis. MRI-DWI examination was after intravenous thrombolysis, and the distribution of the infarct change trend was not statistically significant.

**Table 1:** Risk factors of DWI reversal and enlargement in patients with cerebral infarction and grouping features of relevant experimental examination.

Feature factors	Total	DWI-reversal (*n* = 48)	DWI-enlargement (*n* = 51)	*p-*value
Age (year) median (IQR)	67.00 [58.00, 1.50]	65.00 [54.75, 69.25]	68.00 [58.00, 74.00]	0.089
Sex, *n* (%)	72 (72.7)	36 (75.0)	36 (70.6)	0.79^*^
Risk factors, *n* (%)				
Hypertension	64 (64.6)	27 (56.2)	37 (72.5)	0.138
Diabetes	24 (24.2)	12 (25.0)	12 (23.5)	1
Hyperlipidemia	16 (16.2)	8 (16.7)	8 (15.7)	1
Coronary disease	6 (6.1)	1 (2.1)	5 (9.8)	0.235
Smoking	67 (67.7)	31 (64.6)	36 (70.6)	0.672
Drinking	16 (16.2)	8 (16.7)	8 (15.7)	1
Stroke history, *n* (%)	7 (7.1)	0 (0.0)	7 (13.7)	0.023^*^
Laboratory examination				
Low-density lipoprotein (mmol/l)	3.02 (0.91)	2.80 (0.84)	3.22 (0.93)	0.028^*^
Homocysteine (HCY)	16.04 (11.96)	17.22 (14.86)	14.96 (8.52)	0.374
Glycosylated hemoglobin (%)	6.88 (2.01)	6.98 (2.25)	6.78 (1.76)	0.647
Fasting blood glucose (mmol/L)	6.69 (2.96)	6.62 (3.25)	6.76 (2.70)	0.816
Serum creatinine (μmol/L)	80.16 (26.68)	80.27 (31.86)	80.06 (21.08)	0.97
Carbamide (mmol/L)	4.84 (1.84)	5.12 (2.28)	4.58 (1.28)	0.161
Uric acid (μmol/L)	304.87 (98.64)	299.49 (94.97)	309.89 (102.75)	0.622
International normalized ratio	0.97 (0.12)	0.95 (0.16)	0.99 (0.06)	0.165
Fibrinogen	2.60 (0.73)	2.54 (0.90)	2.65 (0.55)	0.51
Platelet (PLT)	206.80 (61.79)	202.57 (57.90)	210.85 (65.69)	0.528

^*^Indicating a significant difference.

**Table 2:** Clinical-related grouping features of DWI reversal and DWI enlargement in patients with cerebral infarction.

Feature factors	Total	DWI-reversal (*n* = 48)	DWI-enlargement (*n* = 51)	*P-*value
NIHSS score at admission (median [IQR])	4.00 [2.00, 6.00]	4.00 [2.00, 6.25]	4.00 [3.00, 5.50]	0.816
Thrombolytic rate (%)	19 (19.2)	10 (20.8)	9 (17.6)	0.883
NIHSS score after thrombolysis (median [IQR])	3.00 [0.00, 5.25]	2.00 [0.00, 4.00]	3.50 [0.25, 5.75]	0.471
NIHSS score at discharge (median [IQR])	3.00 [2.00, 6.00]	2.00 [1.00, 4.25]	4.00 [2.00, 7.00]	0.007^*^
ΔNIHSS	−0.63 (2.60)	−1.56 (2.78)	0.25 (2.09)	<0.001^*^
mRS score at discharge (median [IQR])	2.00 [1.00, 3.00]	2.00 [1.00, 3.00]	3.00 [2.00, 4.00]	0.001^*^
mRS score at 90 days (median [IQR])	1.00 [0.00, 3.00]	0.00 [0.00, 2.00]	1.00 [0.00, 3.00]	0.024^*^
ΔmRS	−0.90 (1.54)	−0.77 (1.75)	−1.02 (1.30)	0.423
Time from onset to MR examination (h)	37.2 ± 16.1	35.6 ± 14.6	38.8 ± 16.3	0.268
Infarction volume	16011.12 (26807.22)	12894.33 (21523.70)	18944.57 (30901.99)	0.264
DWI-ASPECTS at admission (median [IQR])	2.00 [2.00, 4.00]	2.00 [1.00, 3.00]	3.00 [2.00, 6.00]	0.026^*^

DWI-ASPECTS, diffusion-weighted imaging – Alberta early stroke CT Score; mRS, modified ranking score; n, quantity; NIHSS, National Institutes of Health Stroke Scale; Δ NIHSS, discharged NIHSS – hospitalized NIHSS. ΔmRS = 90-day mRS score – discharge mRS score; ^*^Indicates a significant difference.

Besides differences in the laboratory indexes, including fasting blood glucose (*p =* 0.018), urea (*p =* 0.014), platelets (*p* = 0.016), DWI-ASPECT score (*p =* 0.038), there were no significant differences in the distribution of the primary features of the training and the test set between the two groups.

There was no significant difference between the basic infarct volume (*p =* 0.408) and mRS at 90 days between the training set and the test set (*p =* 0.628) (Tables 3, 4).

**Table 3:** Risk factors of the training set and the test set in patients with cerebral infarction and grouping features of relevant experimental examination.

Feature factors	Total	Training set (*n* = 66)	Test set (*n* = 33)	*P*-value
Age (year)	67.00	67.00	63.00	0.42
Median (IQR)	[58.00, 71.50]	[58.00, 73.75]	[58.00, 70.00]	
Sex, *n* (%)	72 (72.7)	45 (68.2)	27 (81.8)	0.231
Risk factors, *n* (%)				
Hypertension	64 (64.6)	44 (66.7)	20 (60.6)	0.71
Diabetes	24 (24.2)	19 (28.8)	5 (15.2)	0.214
Hyperlipidemia	27 (27.3)	17 (25.8)	10 (30.3)	0.811
Coronary disease	6 (6.1)	5 (7.6)	1 (3.0)	0.655
Smoking	67 (67.7)	48 (72.7)	19 (57.6)	0.197
Drinking	16 (16.2)	10 (15.2)	6 (18.2)	0.923
Stroke history, *n* (%)	7 (7.1)	5 (7.6)	2 (6.1)	1
Laboratory examination				
Low-density lipoprotein (mmol/l)	3.02 (0.91)	3.09 (0.84)	2.87 (1.02)	0.28
Homocysteine (HCY)	16.04 (11.96)	15.39 (9.51)	17.26 (15.71)	0.484
Glycosylated hemoglobin (%)	6.88 (2.01)	7.23 (2.09)	6.18 (1.66)	0.018^*^
Fasting blood glucose (mmol/L)	6.69 (2.96)	7.16 (3.25)	5.83 (2.09)	0.043
Serum creatinine (μmol/L)	80.16 (26.68)	81.65 (27.61)	77.29 (24.99)	0.463
Carbamide (mmol/L)	4.84 (1.84)	5.18 (1.96)	4.19 (1.39)	0.014^*^
Uric acid (μmol/L)	304.87 (98.64)	303.46 (100.80)	307.63 (95.88)	0.852
International normalized ratio	0.97 (0.12)	0.98 (0.06)	0.95 (0.19)	0.264
Fibrinogen	2.60 (0.73)	2.68 (0.76)	2.43 (0.66)	0.142
Platelet (PLT)	206.80 (61.79)	217.85 (55.07)	184.70 (69.22)	0.016^*^

^*^Indicating a significant difference.

**Table 4:** Clinical-related grouping characteristics of the training set and the test set in patients with cerebral infarction.

Feature factors	Total	Training set (*n* = 66)	Test set (*n* = 33)	*P*-value
NIHSS score at admission (median [IQR])	4.00 [2.00, 6.00]	4.00 [2.25, 6.00]	5.00 [2.00, 6.00]	0.86
Thrombolytic rate (%)	19 (19.2)	13 (19.7)	6 (18.2)	1
NIHSS score after thrombolysis (median [IQR])	3.00 [0.00, 5.25]	3.50 [0.25, 5.75]	2.00 [0.00, 4.00]	0.471
NIHSS score at discharge (median [IQR])	3.00 [2.00, 6.00]	3.50 [2.00, 6.00]	3.00 [2.00, 6.00]	0.843
ΔNIHSS	−0.63 (2.60)	−0.68 (2.82)	−0.52 (2.12)	0.765
mRS score at discharge (median [IQR])	2.00 [1.00, 3.00]	2.50 [2.00, 3.00]	2.00 [1.00, 3.00]	0.27
mRS score at 90 days (median [IQR])	1.00 [0.00, 3.00]	1.00 [0.00, 3.00]	1.00 [0.00, 2.00]	0.628
ΔmRS	−0.90 (1.54)	−0.92 (1.33)	−0.85 (1.91)	0.818
Time from onset to MR examination (h)	37.2 ± 16.1	38.69 ± 14.36	36.50 ± 15.77	0.675
Infarction volume	16011.12 (26807.22)	14423.82 (28410.70)	19185.73 (23359.07)	0.408
DWI-ASPECTS at admission (median [IQR])	2.00 [2.00, 4.00]	2.00 [1.25, 3.00]	4.00 [2.00, 5.00]	0.038^*^

DWI-ASPECTS, diffusion-weighted imaging – Alberta early stroke CT Score; mRS, modified Rankin Score; n, quantity; NIHSS, National Institutes of Health Stroke Scale; Δ NIHSS, discharged NIHSS – hospitalized NIHSS. ΔmRS, 90-day mRS score – discharge mRS score; ^*^Indicates a significant difference.

### Performance comparison of prediction models

When modal images are included, the model has a different prediction performance. The AUC of the SWI + CTP comprehensive model was 0.952, ACC was 0.909, Sen was 0.889, and SPE was 0.933. The prediction efficiency was the highest. Compared with the value of AUC, the SWI model was 0.874, and the CTP model was 0.715 (Table 5).

**Table 5:** Performance of different prediction models.

Models	AUC	ACC	SEN	SPE	PPV	NPV
SWI	0.874	0.848	0.889	0.800	0.842	0.857
CTP	0.715	0.727	0.778	0.667	0.735	0.714
SWI + CTP	0.952	0.909	0.889	0.933	0.941	0.875

**Figure 4:**
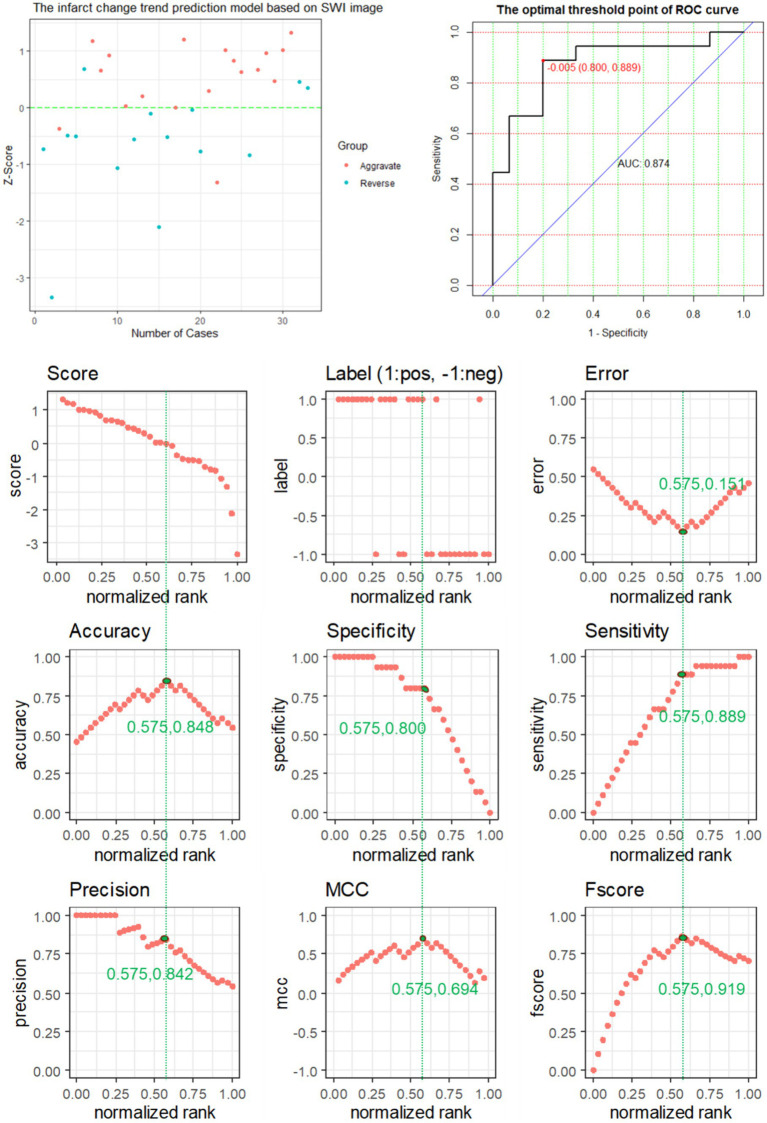
The predictive power of SWI model features for predicting the changing trend of infarction.

The performance of the SWI prediction model is evaluated, and its evaluation metrics are at the optimum when the value of the standardization level is equal to 0.575: ACC of 0.848 reaches the highest value, ERR of 0.151 is the lowest value, the accuracy rate of 0.842 is relatively high, MCC of 0.694 is the highest value, and F score of 0.919 is the highest value (Figure 4).

**Figure 5:**
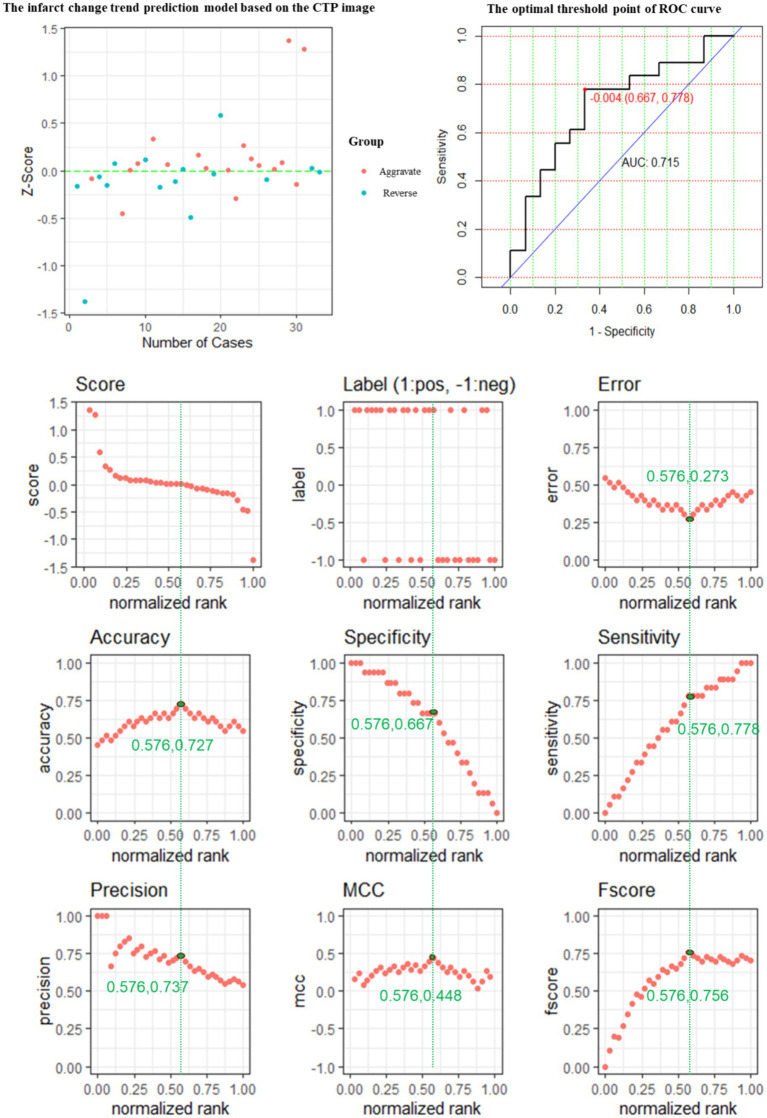
The predictive power of the CTP model features for predicting the changing trend of infarction.

The performance of the CTP prediction model is evaluated, and its evaluation metrics are at the optimum when the value of the standardization level is equal to 0.576: ACC is 0.727, reaching the highest value, ERR is 0.273, reaching the lowest value, and the accuracy rate is 0.737, a relatively high value, MCC of 0.449 is the highest value, and *F*-score of 0.756 is the highest value (Figure 5).

**Figure 6:**
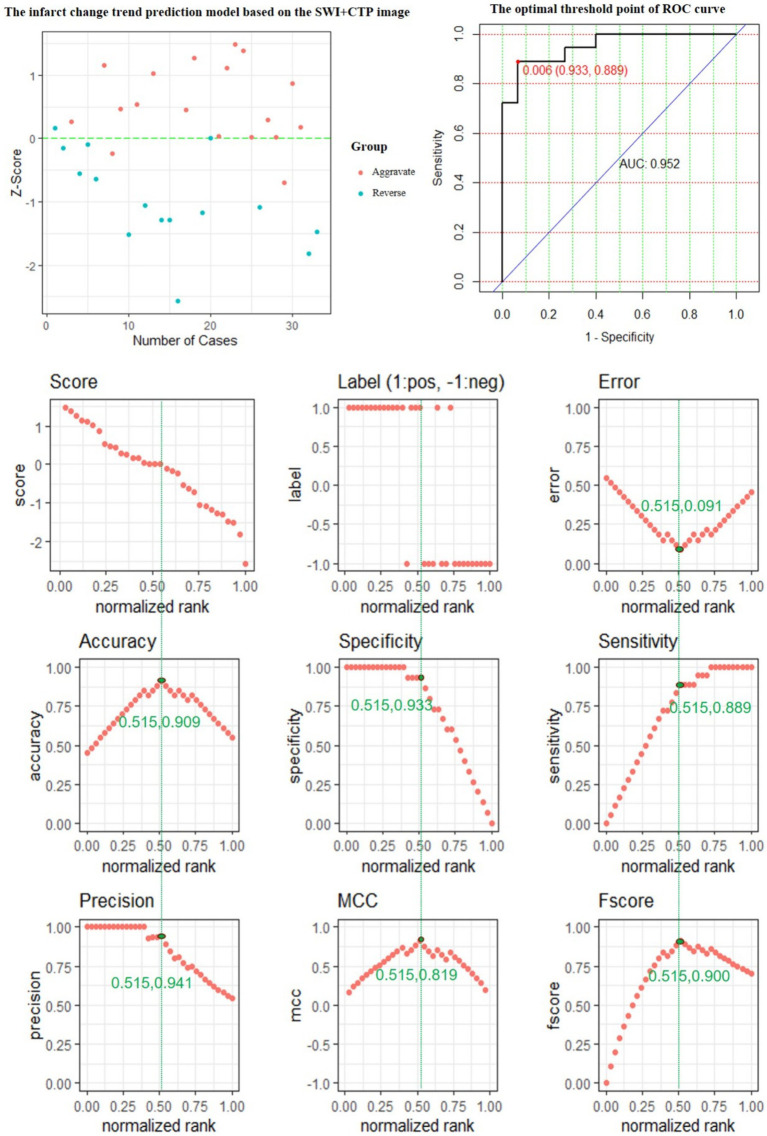
The predictive power of the SWI + CTP model features for predicting the changing trend of infarction.

The performance of the CTP + SWI prediction model is evaluated, and its evaluation metrics are at the optimum when the value of the standardization level is equal to 0.515: ACC is 0.909, reaching the highest value; ERR is 0.091, reaching the lowest value; accuracy is 0.941, a relatively high value; MCC is 0.819, reaching the highest value; and *F* score is 0.900, reaching the highest value (Figure 6).

## Discussion

There is a correlation between venous reflux disorder and perfusion defect of SWI image signal. CTP contains perfusion information in space and time. There is a complementary basis between them. By establishing a comprehensive model to predict the changing trend of infarction volume in patients with cerebral infarction, the prediction efficiency has improved, proving the complementarity of the two images. It verifies the correlation between the combination of the two image features and the changing trend and infarction prognosis.

Although CTP is currently superior to SWI in determining ischemic penumbra, SWI is increasingly proving to be a helpful imaging sequence for evaluating acute stroke. Because of its lack of contrast and the absence of radiation associated with CT, as well as its reliability in detecting ischemic penumbra, it has increasingly become a powerful tool for detecting ischemic penumbra in the treatment and management of acute cerebral infarction (Abu-samra et al., 2021).

SWI is a less time-consuming technique that does not require a contrast agent and can be considered an alternative to CTP and MRP. SWI has great value in detecting penumbra and helping to guide patients quickly for thrombolytic therapy (Kao et al., 2012; Liu et al., 2016). SWI can provide a metabolic examination capability similar to The Mean Transit Time (MTT) (Jiang et al., 2021) and correlate with other perfusion indicators (Luo et al., 2017). Further research suggests that SWI-DWI mismatch and DWI-PWI (MTT) mismatch have similar effects (Dejobert et al., 2016; Wang et al., 2017).

Some studies have used SWI to predict changes in infarction volume, and mismatched SWI-DWI and MTT-DWI can predict infarction enlargement (Kao et al., 2012). But the study had a limited number of patients, mainly because the imaging studies were incomplete. What’s more, the ASPECTS system is used, which has certain subjective score differences. Moreover, the ASPECTS system is insensitive to subtle infarction changes. Our study can solve the subjectivity of visual evaluation using image radiomics methods and improve accuracy.

Other studies suggest that SWAN’s quantitative evaluation of low signal areas in medullary or cortical veins can provide information on venous reflux and can be used to predict infarction growth in patients with non-reperfusion large artery occlusion (Yamaguchi et al., 2018). At the same time, some studies have tried to quantify the low signal area in a medullary or cortical vein by measuring the number of pixels for the evaluation of reperfusion treatment and achieved effective results (Lou et al., 2014). To avoid the influence of subjective factors, we further use imaging radiomics in our study to more simply and effectively predict the changes of infarction.

The CTP is a four-dimensional spatiotemporal image that contains features of both spatial and temporal entities (Giacalone et al., 2018). The time signal of the original perfusion image has demonstrated the ability to predict the lesion evolution of cerebral infarction, and the time information also plays a role in quantifying collateral blood flow (Kim et al., 2014). Firstly, the perfusion information of each VOI in the original perfusion CT image was numerically simulated. Then the spatial information of VOI and adjacent voxels was expressed in image brightness and texture features. The spatial information was incorporated through the graphical features, and the changing trend of infarction was directly predicted using these extracted image features, which completes the integration of temporal and spatial information of blood flow into the prediction model.

Although CTP plays an important role in mismatch detection, it is not as good as SWI in predicting infarction changes because vascular hydrodynamics needs a complex modeling process. To simplify the model, we first assumed that the blood flow in the blood vessel is uniform and stable. On this premise, we established a perfusion index model. Although fluid dynamics may not have a significant impact based on perfusion (time density curve), the lack of blood flow factors (especially venous reflux factors) will affect the model’s performance and ultimately affect the prediction results. The SWI model can predict the changes of infarction more accurately because it provides venous reflux information.

Compared to the perfusion indicator feature model, the SWI feature model used fewer indicator features while obtaining higher classification prediction performance, suggesting to some extent the importance of the correlation of venous return in prediction. Additionally, SWI is proficient at reflecting impediments in venous outflow and ischemic areas, which are highly correlated with the trajectory of infarct progress. Therefore, the sensitivity of the SWI model was as high as that of the SWI + CTP model. Of course, combining the two features to form the model gives better results, suggesting the complementary nature of the two to some extent. The cerebral vasculature can be considered a complex system of large and microvascular circulation, including large and small arteries, capillaries, and large and small veins. This complex cerebral vascular structure, hemodynamics, and tissue perfusion may significantly affect the function of the adjacent brain parenchyma, affecting the infarction changes (Liebeskind, 2015; Liu et al., 2018). Thus, the complex combination of perfusion and venous return changes has predictive value for infarction changes.

Of course, adding spatiotemporal information will inevitably lead to a massive increase in data. Therefore, we selected the image information of the adjacent cerebral infarction area and obtained a more accurate prediction performance by aggregating local space and time, consistent with some studies (Giacalone et al., 2017). Using regional image patches instead of the single-voxel method can predict cerebral infarction tissue fate and reduce data redundancy. At the same time, we used the feature screening method of sparse representation to analyze the correlation between multi-dimensional complex data of regional image patches. Select key features with spatiotemporal information to clarify the law of changes in the infarction and surrounding areas, in which the spatial information was included through the image screening features, and then the screened features were interpreted reverse. It provides a preliminary basis for the later clinical application.

In this study, although nearly 500 patients were included in the data collection process in the early stage, due to the poor accessibility of MRI examination, the modality was incomplete or could not be rechecked according to the process, and the influence of imaging quality, patient tolerance, and other factors, only about 100 patients was finally included in the study, resulting in a relatively small number of cases in the current study.

The difficulty in collecting image data (especially multimodal MRI) in the process of data inclusion is because the role of image modality in stroke diagnosis and treatment is not very clear, which leads to clinicians’ insufficient understanding of the importance of image data in stroke diagnosis and treatment guidance and prediction. Therefore, a thorough image examination scheme has not been formed to standardize the clinical stroke diagnosis and treatment.

Based on the current research results, we will gradually standardize the imaging examination process for stroke in the later stage. Subsequently, we will further expand the data set and enrich the experimental verification to improve the reliability and robustness of the model to obtain more convincing results. In addition, the single-center data experiment also has certain limitations. In the later stage, we will collect multi-center data, constantly improve the feature extraction, feature screening, and classification model technology, and iterate to improve the model effect.

Notably, our current results indicate that a portion of patients outside the time window experienced infarct reversal, and these changes could be predicted based on imaging features reflecting the complex interplay between perfusion and venous return alterations, which hold predictive value for infarct progression. To some extent, this suggests that interventions tailored to each patient’s specific perfusion and venous return status might pave the way for exploring effective treatment approaches beyond the established time window. Investigating potential therapeutic avenues for the post-window period is an area we may explore in future studies.

## Data availability statement

The datasets presented in this article are not readily available because the data that support the findings of this study are available on request from the corresponding author. The data are not publicly available due to privacy or ethical restrictions. Requests to access the datasets should be directed to linjixian@fudan.edu.cn.

## Ethics statement

The study was registered in Chinese Clinical Trial Registry (Registration number: ChiCTR2100045753). Ethical approval for this study was obtained from the institutional review board at Minhang Hospital, Fudan University, Shanghai, China. The need for consent for this study was waived by the institutional review board (the institutional review board at Minhang Hospital, Fudan University, Shanghai, China) due to its design.

## Author contributions

GW: Conceptualization, Formal analysis, Funding acquisition, Investigation, Methodology, Resources, Software, Supervision, Validation, Visualization, Writing – original draft. HW: Conceptualization, Data curation, Methodology, Writing – original draft. XM: Data curation, Formal analysis, Writing – review & editing. HL: Data curation, Formal analysis, Methodology, Writing – original draft. BS: Data curation, Methodology, Writing – original draft. JZ: Conceptualization, Writing – review & editing. XW: Conceptualization, Data curation, Writing – review & editing. JL: Conceptualization, Data curation, Formal analysis, Funding acquisition, Investigation, Methodology, Validation, Writing – original draft, Writing – review & editing.

## Footnotes

^1^https://mirrors.sjtug.sjtu.edu.cn/cran/web/packages/tableone/index.html
