# Supplementary material for: SWI and CTP fusion model based on sparse representation method to predict cerebral infarction trend
Source: Front Neurosci. 2024 Jun 20;18:1360459. doi: 10.3389/fnins.2024.1360459 (PMC11222412; doi:10.3389/fnins.2024.1360459)
Supplement: Supplementary file 1 [file Data_Sheet_1.docx]

**SUPPLEMENTAL MATERIAL**

**Supplement****al Methods**

**Feature extraction method based on image patch sparse representation**

We first expanded the DWI segmented image in the previous section by 20 pixels outside the lesion area and matched the segmentation results of SWI images on the corresponding interface. Then, 513 gray features, texture, and wavelet features are extracted from the SWI image. Among them, 18 gray features are extracted (gray features describe the statistical distribution of voxel intensity in the infarction and surrounding areas). 39 texture features (texture features quantify the spatial arrangement of infarct and surrounding area) were extracted and divided into 4 subgroups. There are 8 second-order texture features, which are based on gray level co-occurrence matrix (GLCM); There are 31 high-order texture features, including 13 based on gray level run length matrix (GLRLM), 13 based on gray level size zone matrix (GLSZM), and 5 based on neighborhood gray-tone difference matrix (NGTDM). Wavelet feature results from texture feature quantized by wavelet transform, including intensity features and texture features of different frequency bands. All possible combinations of wavelet filtering are (LLH, LHL, LHH, HLL, HLH, HHL, HHH, and LLL). High-pass or low-pass filters are applied to decompose in three dimensions, 8 decompositions are generated at each level, and each feature image is decomposed into 8 sub-bands to obtain 456 ((18 + 39) * 8) wavelet features (e-Table 1).

**Collection and formation of perfusion indicator**

**Time-density curve**

CTP image is a series of simultaneous scans of selected layers during contrast injection. Therefore, the time-density Curve (TDC) of each pixel in the layer can be obtained. As shown in e-Figure 4, TTP can be obtained according to the time when the maximum contrast concentration appears, CBF can be calculated according to the maximum rising slope, and CBV can be calculated according to the area under the curve.

**Feature extraction of perfusion index**

High-throughput features of infarction and surrounding areas were extracted from CBV images, CBF images, and TTP images. These features include 18 intensity features (intensity features describe the statistical distribution of voxel intensities in the infarction and surrounding areas) and 39 texture features (texture features quantify the spatial arrangement of the infarction and surrounding areas). The list of features is shown in e-Table 2.

**Segmentation of DWI infarction core area**

The changes in infarction can be divided into infarction enlargement and infarction reversal. Infarction enlargement refers to the area where there is no high signal on baseline DWI imaging, and the histopathological changes of infarction appear in the later stage. Infarction area reversal refers to the reversal of histopathological changes of neuronal damage in DWI high signal area after blood flow reperfusion. According to the initial examination and re-examination results, each patient was divided into the infarction reversal group and infarction enlargement group according to the outcome of infarction changes (e-Figure 2). The infarction core area was segmented according to the results of two MRI-DWI.

Segmentation method: the infarction core (if any) was manually segmented by two neurologists using open-source DICOM-viewing software (ITK snap, http://www.itksnap.org/pmwiki/pmwiki.php ）on *b* = 1000 s / mm^2^ DWI images, and the tissue was determined as infarction according to the high signal compared with the surrounding tissue and the contralateral hemisphere. The imaging artifacts similar to the infarction core are distinguished from the actual core according to the peripheral information from the whole image, including symmetry/bilaterality, the presence of metal and other pathogenic materials, and the specific anatomical positions near the skull or on the brain, bone and air interface. A parenchymal hematoma with a continuation of the infarction or within the infarction is included in the segmentation. As shown in e-Figure 3: as a result of manual segmentation, red segmentation areas can be seen in the coronal plane, cross-sectional plane, and sagittal plane, respectively, and the infarct volume can be calculated accordingly.

**Feature extraction method based on image patch sparse representation**

We first expanded the DWI segmented image in the previous section by 20 pixels outside the lesion area and matched the segmentation results of CTP and SWI images on the corresponding interface. Then, 513 gray features, texture, and wavelet features are extracted from the DWI image, T2 flair image, and SWI image. Among them, 18 gray features are extracted (gray features describe the statistical distribution of voxel intensity in the infarction and surrounding areas). 39 texture features (texture features quantify the spatial arrangement of infarct and surrounding area) were extracted and divided into 4 subgroups. There are 8 second-order texture features, which are based on gray level co-occurrence matrix (GLCM); There are 31 high-order texture features, including 13 based on gray level run length matrix (GLRLM), 13 based on gray level size zone matrix (GLSZM), and 5 based on neighborhood gray-tone difference matrix (NGTDM). Wavelet feature results from texture feature quantized by wavelet transform, including intensity features and texture features of different frequency bands. All possible combinations of wavelet filtering are (LLH, LHL, LHH, HLL, HLH, HHL, HHH, and LLL). High-pass or low-pass filters are applied to decompose in three dimensions, 8 decompositions are generated at each level, and each feature image is decomposed into 8 sub-bands to obtain 456 ((18 + 39) * 8) wavelet features (e-Table 1).

**Statistical analysis and model evaluation**

**Prediction model statistics**

The performance of the four prediction models was evaluated using the Receiver Operating Characteristic Curve (ROC) to compare the efficacy of the models. Six metrics are also displayed: The Area Under The Corresponding Curve (AUC), ACC, SEN, SPE, Positive Predictive Value (PPV), and Negative Predictive Value (NPV). They are calculated as follows.

$ACC=\frac{TP+TN}{TP+TN+FP+FN}$ (1)

$\text{SEN}=\frac{\text{TP}}{\text{TP}+\text{FN}}$ (2)

$SPE=\frac{TN}{TN+FP}$ (3)

$\text{PPV}=\frac{\text{TP}}{\text{FP}+\text{TP}}$ (4)

$\text{NPV}=\frac{\text{TN}}{\text{FN}+\text{TN}}$ (5)

where True Positive (TP) indicates cases in which infarct enlargement was correctly identified, True Negative (TN) indicates cases in which infarct reversal was correctly identified, and False Positive (FP) indicates cases in which infarct reversal was identified as infarct enlargement. False Negative (FN) indicates cases in which infarct enlargement was identified as infarct reversal.

In the ROC curve, the vertical axis (Y-axis) is the True Positive Rate (TPR, equivalent to Sensitivity), the proportion of positive samples correctly identified as positive. The horizontal axis (X-axis) is a False Positive Rate (FPR, equivalent to 1-specificity), that is, the proportion of negative samples incorrectly identified as positive samples;

$TPR=SEN=\frac{TP}{TP+FN}$ (6)

$FPR=1-TNR=\frac{FP}{TN+FP}=\frac{FP}{N}$ (7)

ERR is defined as the ratio of the misclassified samples to the total samples. Precision rate (or accuracy rate) refers to the percentage of samples identified as positive cases that are true positive cases. Recall rate (R) refers to the percentage of positive cases found in the sample (identified as positive cases), called TPR and SEN.

$Err=\frac{FP+FN}{N_{sample}}$ (8)

$P=\frac{TP}{TP+FP}$ (9)

$R=SEN=\frac{TP}{TP+FN}$ (10)

The F score is the summed average of precision and recall rate; MCC is a metric for evaluating the model, and the different divisions in positive and negative cases do not affect the final results.

$\frac{1}{F_{1}}=\frac{1}{2}(\frac{1}{P}+\frac{1}{R})$ (11)

$MCC=\frac{TP\times TN-FP\times FN}{\sqrt{(TP+FP)(TP+FN)(TN+FN)(TN+FP)}}$ (12)

| **e-Table 1. Image list of high-throughput feature extraction** | | |
| --- | --- | --- |
| Grayscale | 1) energy 2) histogram entropy 3) peak 4) maximum 5) average absolute error 6) average 7) median 8)minimum 9) grayscale range 10) root mean square 11) skew 12) standard deviation 13) histogram uniformity 14) variance 15) histogram mean 16) histogram variance 17) histogram skew 18) histogram peak | 18 |
| Texture | Total number of texture features | 39 |
| GLRLM | 1) energy 2) contrast 3) correlation 4) homogeneity 5) variance 6) sum average 7) entropy 8) dissimilarity | 8 |
| GLSZM | 9) short-run emphasis 10) long-run emphasis 11) gray-level nonuniformity 12) run-length nonuniformity 13) run percentage 14) low gray-level run emphasis 15) high gray-level run emphasis 16) short-run low gray-level emphasis 17) short-run high gray-level emphasis 18) long-run low gray-level emphasis 19) long-run high gray-level emphasis 20) gray-level variance 21) run-length variance | 13 |
| GLSZM | 22) small zone emphasis 23) large zone emphasis 24) gray-level nonuniformity 25) zone-size nonuniformity 26) zone percentage 27) low gray-level zone emphasis 28) high gray-level zone emphasis 29) small zone low gray-level emphasis 30) small zone high gray-level emphasis 31) large zone low gray-level emphasis 32) large zone high gray-level emphasis 33) gray-level variance 34) zone-size variance | 13 |
| NGTDM | 35) coarseness 36) contrast 37) busyness 38) complexity 39) strength | 5 |
| Wavelet | Eight high-frequency and low-frequency components in three directions of wavelet (LLL, HLL, LHL, HHL, LLH, HLH, LHH, and HHH) | 456 |
|  | Total number of features | 513 |

| **e-Table 2. image feature list of high-throughput feature extraction.** | | |
| --- | --- | --- |
| Grayscale | 1) energy 2) histogram entropy 3) peak 4) maximum 5) average absolute error 6) average 7) median 8) minimum 9) grayscale range 10) root mean square 11) skew 12) standard deviation 13) histogram uniformity 14) variance 15) histogram mean 16) histogram variance 17) histogram skew 18) histogram peak | 18 |
| Texture | Total number of texture features | 39 |
| GLRLM | 1) energy 2) contrast 3) correlation 4) homogeneity 5) variance 6) sum average 7) entropy 8) dissimilarity | 8 |
| GLSZM | 9) short-run emphasis 10) long-run emphasis 11) gray-level nonuniformity 12) run-length nonuniformity 13)run percentage 14) low gray-level run emphasis 15) high gray-level run emphasis 16) short-run low gray-level emphasis 17) short-run high gray-level emphasis 18) long-run low gray-level emphasis 19) long-run high gray-level emphasis 20) gray-level variance 21) run-length variance | 13 |
| GLSZM | 22) small zone emphasis 23) large zone emphasis 24) gray-level nonuniformity 25) zone-size nonuniformity 26) zone percentage 27) low gray-level zone emphasis 28) high gray-level zone emphasis 29) small zone low gray-level emphasis 30) small zone high gray-level emphasis 31) large zone low gray-level emphasis 32) large zone high gray-level emphasis 33) gray-level variance 34) zone-size variance | 13 |
| NGTDM | 35) coarseness 36) contrast 37) busyness 38) complexity 39) strength | 5 |
|  | Total number of features | 57 |

**
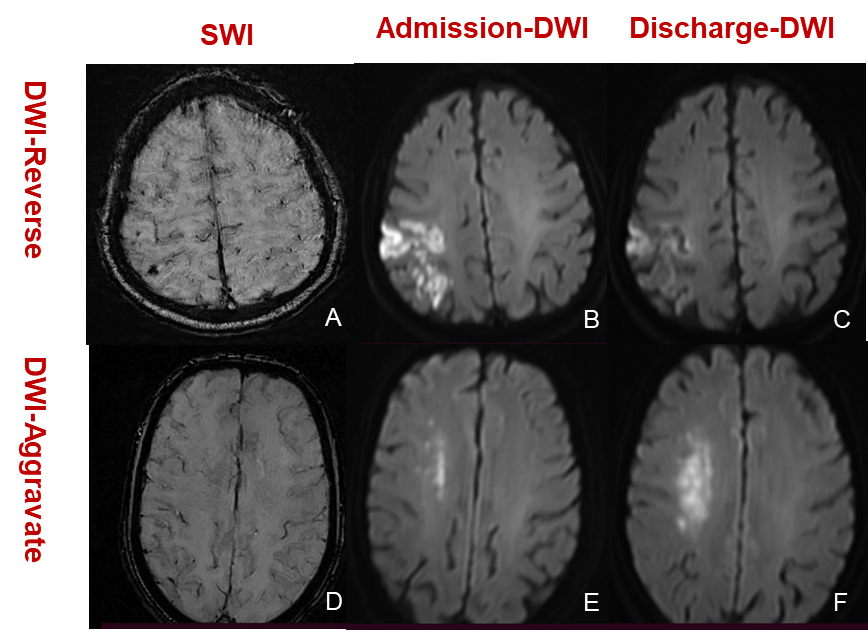
**

**e-Figure 2. Schematic diagram of infarct volume change trend in patients with cerebral infarction**

**
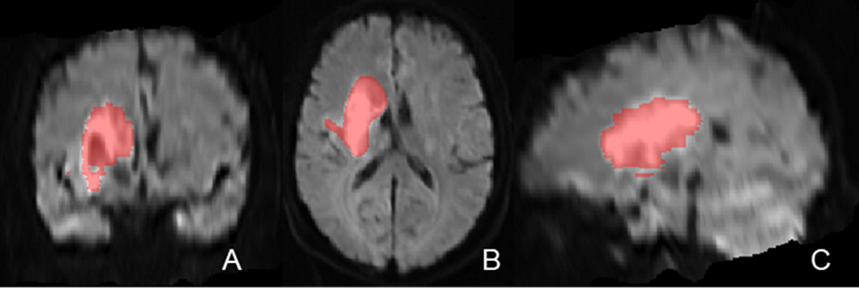
**

**e-Figure 3. DWI image segmentation of infarct volume of a cerebral infarction patient.**

A. Coronal plane; B. Transverse plane; C. Sagittal plane. The segmented area is shown in red.


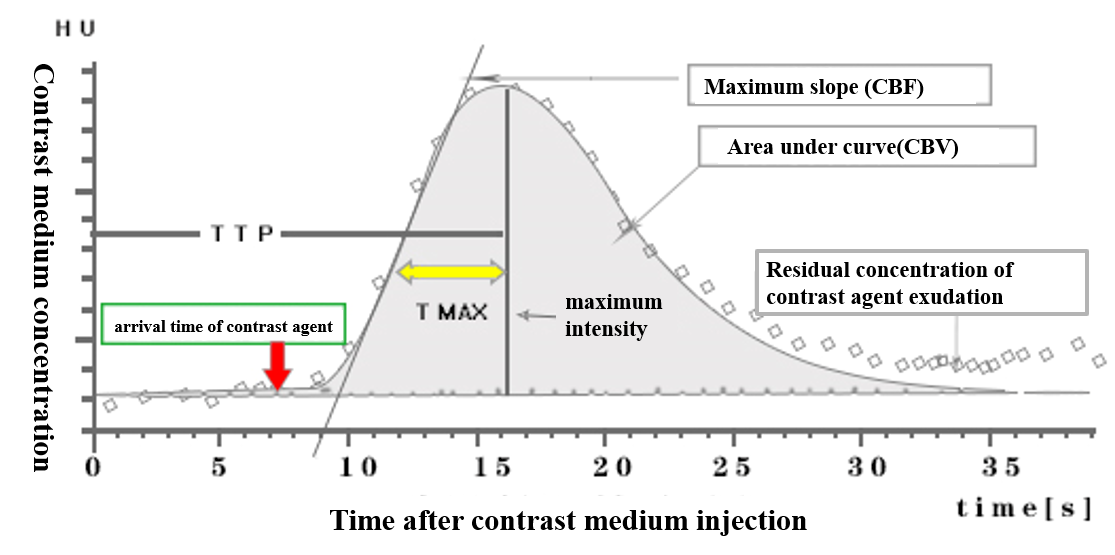


**e-Figure 4. The pattern of time density curve formation**
